# Improvement of the low knowledge, attitude and practice of hepatitis B virus infection among Saudi national guard personnel after educational intervention

**DOI:** 10.1186/1756-0500-5-597

**Published:** 2012-10-30

**Authors:** Majid S Al-Thaqafy, Hanan H Balkhy, Ziad Memish, Yahya M Makhdom, Adel Ibrahim, Abdulfattah Al-Amri, Abdulhakeem Al-Thaqafi

**Affiliations:** 1 Infection Prevention and Control, King Abdulaziz Medical City, Jeddah, Saudi Arabia; 2 Infection Prevention and Control, King Abdulaziz Medical City, Riyadh, Saudi Arabia; 3 Deputy Minister of Health for Public Health, Ministry of Health, Riyadh, Saudi Arabia; 4 Family and Community Medicine, Ministry of Health, Jeddah, Saudi Arabia; 5 Statistical Department, Primary Healthcare, Ministry of Health, Jeddah, Saudi Arabia; 6 Pathology & Laboratory Medicine, King Abdulaziz Medical City, Jeddah, Saudi Arabia

**Keywords:** Hepatitis B virus, Knowledge, Attitude and Practice, Military, Saudi Arabia

## Abstract

**Background:**

Although the risk of hepatitis B virus (HBV) was reported to be higher in military personnel than the general population in Saudi Arabia (SA), there is lack of studies assessing HBV awareness among them. The objective was to evaluate the knowledge, attitude and practice (KAP) of HBV infection among military personnel.

**Methods:**

An intervention design with pre- and post-education KAP questionnaire was completed among National Guard soldiers working in Jeddah during January 2009. Educational intervention was provided through educational leaflets, group and individual discussions, visual show, and a lecture. A score was created from the correct answers to 58 questions.

**Results:**

A total of 400 male soldiers with mean age 30.7 ± 6.1 years completed both questionnaires. The majority had school education (96.8%) and in the lower military ranks (66.0%). Only 19.5% of soldiers reported HBV vaccine intake. The low median and inter-quartile range of the pre-intervention score (16, 6–26) markedly increased after education (to 53, 50–55, p<0.001). The overall improvement of mean KAP score (204%) was also observed in all its component scores; disease nature (272%), methods of transmission (206%), prevention and control (109%), attitude (155%), and practice (192%). The improvement was evident irrespective of socio-demographic characteristics and history of HBV vaccine. KAP scores were significantly associated with higher educational levels, higher monthly income, administrative jobs, and higher job ranks.

**Conclusion:**

We are reporting a low level of HBV awareness among Saudi military population. The study confirms the need and effectiveness of focused multifaceted educational campaigns among the military population.

## Background

Viral hepatitis, especially those caused by HBV, represents a worldwide significant cause of morbidity and mortality [1,2]. In SA, viral hepatitis represents a major public health problem. SA used to be among the countries which had the highest endemicity of seropositive hepatitis B surface antigen (HBsAg) [3]. Despite implementation of a childhood HBV vaccination program in 1990, HBV is currently reported more frequently among Saudi than the hepatitis C virus [4]. Moreover, according to the Saudi Ministry of Health (MOH) data, viral hepatitis was ranked the second most common reportable viral disease after chickenpox, with more than 5000 new cases diagnosed in 2009 [4]. Jeddah, a multicultural coastal city, reported new HBV infections, the risk level being 50% higher than any other city in SA [4].

Lack of awareness of the risk of HBV and its consequences are recognized as a major deterrent to immunization among HBV high risk groups [5]. The incidence and prevalence HBV seropositivity ranked the second reportable communicable disease among SA National Guard (SANG) personnel in all regions and the first among SANG personnel in Jeddah [6]. Although the risk of HBV seropositivity was reported to be higher in the military population than the general population of SA [7,8] and elsewhere [9,10], and despite the fact that HBV seropositivity in SA was one of the highest in the world, [3] there was a definite lack of studies assessing KAP among military personnel of SA. However, there is a couple of studies examining awareness of HBV in SA among dental professionals [11] and patients [12]. Moreover, it appeared that the non-professional Saudi population had little or no knowledge of HBV [12]. The objective of this study was to evaluate the KAP of HBV infection before and after an educational intervention among SANG soldiers.

## Methods

### Population

The current study was conducted among SANG soldiers in Jeddah, SA. Jeddah governorate is located within the Western Region of SA and is considered the main Saudi seaport and, the main entrance for Makkah pilgrims. The population of Jeddah in 2009 was estimated at 3.3 million including 1.5 million non-Saudi [4]. Of the Saudi population, there was an estimated 10,000 male SANG soldiers serving in Jeddah. The population receives health services mainly by the Health Affairs of National Guard who provide one tertiary hospital and five primary healthcare centers.

### Study design

An intervention design with pre- and post-education assessment of KAP of HBV infection was completed among the male SANG soldiers working in Jeddah during January 2009. Four hundred participants were randomly selected from the SANG soldiers roster and the consent form was signed after a verbal explanation of the study objectives. The participation rate was 96% and all participants completed both pre- and post-intervention questionnaires. The KAP of HBV infection was assessed using a structured questionnaire completed immediately before and after the educational intervention. The study design obtained all required ethical approvals from the ethical committees of King Abdulaziz Medical Cities in Jeddah and Riyadh.

### Questionnaire

The self-administered questionnaire was developed to assess the KAP of HBV infection among the study participants and the questions were based on previous surveys with similar objectives [13,14]. Additionally, the validity of the questionnaire was tested after Arabic translation. The lexical equivalence of questionnaire was examined after back translation**.** Content validity was established by a panel of experts from infectious disease medicine, public health, community medicine, epidemiology, biostatistics, and health education. The final version of the questionnaire had a good indicator for reliability (as indicated by Alpha Cronbach test value of 0.82). Sections of the administered questionnaire included identification data, personal and socio-demographic characteristics, general health status, and KAP of HBV infection which included 58 questions.

### Educational intervention

It was established according to the standard principles of designing and implementing a health education program [15]. The content of the educational intervention included standard information pertaining to risk, transmission, and protection of HBV as described by the World Health Organization and the US Centers for Disease Control and Prevention [2,5,16]. The educational intervention was provided through (1) Distributing educational leaflets (including HBV facts, risk factors, hazard to population, misconceptions and misunderstanding of HBV, prevention tools and how to deal with an infected person), (2) Guiding group discussions followed by individual instructor-participant discussions, (3) Attending mini-exhibition round and visual show, and (4) Presenting a PowerPoint lecture titled “Know and Participate to prevent HBV”. The intervention was carried over 20 days with one session per day. Each session lasted 3 hours and included only 20 new participants to allow for personal communication and open discussion.

### Statistical analysis

Data were presented using descriptive statistics in the form of frequencies and percentages for categorical data, and median and inter-quartile range (IQR) for continuous data. Overall KAP score was the sum of correct responses to the 58 questions. One point was given for the correct answer and zero for the wrong answer. Components’ scores included knowledge of HBV disease nature (19 questions), transmission (25 questions), prevention and control (6 questions), attitude (3 questions), and practice (5 questions). KAP score and its components’ scores were treated as non-parametric data and compared between pre- and post-intervention using Wilcoxon signed rank test. Both pre- and post-intervention KAP scores and their components’ scores were compared across socio-demographic characteristics using Mann–Whitney test or Kruskal Wallis test, as appropriate. Qualitative variables were compared using chi-square test or Fisher exact test as appropriate. All P-values were two-tailed. P-value <0.05 was considered as significant. SPSS software (release 16.0, SPSS Inc., Chicago, U.S.) was used for all statistical analyses.

## Results

A total of 400 male soldiers were examined in the current study as shown in Table 1. The mean age was 30.7 ± 6.1 years (median 29 and IQR, 26–34; range 21–48 years). Few soldiers could not read and write (1.0%) or had university degrees (2.2%) while the majority of the soldiers (96.8%) had school education typically secondary education (43.5%). The majority of the soldiers (78.5%) were married. In addition, the majority of soldiers (81.0%) reported monthly income between 3000 and 8000 Saudi Riyals with 61.5% describing their income as sufficient. Almost two-thirds (63.8%) of the soldiers were engaged in technical jobs while around one-third (36.2%) were engaged in administrative jobs. The majority (66%) of soldiers were in the lower military ranks (lower than Corporal). The average service duration at SANG was 10.0 ± 6.8 years (median 9 and IQR, 4–15). Only 19.5% of soldiers reported previous HBV vaccine intake. One-third (35.2%) of the soldiers claimed they had some knowledge of HBV. The most common sources reported for such knowledge were TV/Radio (39.7%), friends (36.9%), and newspapers and magazines (26.9%).

**Table 1:** Overall knowledge, attitude and practice (KAP) scores before and after educational intervention by socio-demographic characteristics among SANG soldiers (Jeddah, 2009)

**Characteristics**	**N (%) mean±SD**	**Overall KAP score**	**Post-intervention Median (IQR)**	p-value*
		**Pre-intervention Median (IQR)**		
**Overall**	400 (100.0%)	16 (6–26)	53 (50–55)	<0.001
**Age** (mean±SD, years)	30.7±6.1			
**Age groups**		P**=0.086	P**=0.107	
<25 years	57 (14.3%)	15 (4.5-25.5)	54 (50.5-55)	<0.001
25-34 years	256 (64.0%)	15 (6–24)	53 (51–54)	<0.001
35+ years	87 (21.8%)	19 (8–30)	52 (49–54)	<0.001
**Educational level**		P**=0.007	*P<0.001*	
Elementary or lower	83 (20.8%)	14 (5–21)	50 (45–53)	<0.001
Intermediate	134 (33.5%)	14.5 (6–22.3)	53 (50–55)	<0.001
Secondary or higher	183 (45.8%)	19 (6–30)	54 (52–55)	<0.001
**Marital status**		P**=0.358	P**=0.284	
Single or divorced	86 (21.5%)	14.5 (5–25.3)	52.5 (50–54)	<0.001
Married	314 (78.5%)	16.5 (6–26)	53 (50–55)	<0.001
**Monthly income, amount**		P**=0.001	P**=0.004	
≤8000 SR	324 (81.0%)	15 (5–24)	53 (50–54)	<0.001
>8000 SR	76 (19.0%)	20.5 (10.5-31)	54 (52–55)	<0.001
**Monthly income, sufficiency**		P**=0.338	P**=0.228	
Sufficient	246 (61.5%)	16 (5–26)	53 (50–55)	<0.001
Insufficient	154 (38.5%)	17 (7–26.3)	53 (49–54)	<0.001
**Type of the job**		P**=0.005	P**=0.026	
Technical	255 (63.8%)	14 (5–24)	53 (49–54)	<0.001
Administrative	145 (36.2%)	19 (9–28)	53 (51–55)	<0.001
**Rank**		P**=0.026	P**=0.007	
Soldier	138 (34.5%)	14 (4.8-23.3)	53 (49–54)	<0.001
First soldier	126 (31.5%)	15.5 (6–24.3)	52.5 (50–54)	<0.001
Corporal or higher	136 (34.0%)	19.5 (7.3-29.8)	54 (51–55)	<0.001
**Service years** (mean±SD)	10.0±6.8			
**Duration of service groups**		P**=0.542	P**=0.123	
<5 years	102 (25.5%)	15.5 (5–26.3)	53 (51–55)	<0.001
5-9 years	125 (31.3%)	15 (6–24.5)	53 (51–55)	<0.001
≥10 years	173 (43.3%)	18 (6.5-27)	53 (49–54)	<0.001
**History of HBV vaccine**		P**=0.039	P**=0.081	
No or don’t know	322 (80.5%)	15 (5–26)	53 (50–54)	<0.001
Yes	78 (19.5%)	19 (9.8-27.8)	53.5 (51–55)	<0.001

N (%), frequency and percentage; Mean±SD, mean and standard deviation; IQR, inter-quartile range; SR, Saudi Riyals.

*Wilcoxon signed rank test ** Mann–Whitney test or Kruskal Wallis test as appropriate.

A significant (p<0.001) increase in the frequency of correct answers was shown in all general knowledge questions after educational intervention (Table 2). Those who correctly identified the infectious nature of HBV increased from 33.3% before intervention to 97.8% after intervention. Those who correctly recognized the possibility of developing complications in HBV patients increased from 30.3% before intervention to 94.5% after intervention. Those who correctly acknowledged major methods of HBV transmission as blood and sexual intercourse increased from 58.0% and 39.8%, respectively before intervention to 99.8% and 99.5%, respectively after intervention. Those who correctly recognized the possibility of infected dental and surgical tools to transmit HBV increased from 42.8% and 47.5%, respectively before intervention to 99.5% and 99.3%, respectively after intervention. Those who were aware of the availability of vaccine against HBV increased from 50.5% before intervention to 96.5% after intervention.

**Table 2:** Frequency of correct answers for individual knowledge, attitude, and practice (KAP) questions of the pre- and post-intervention questionnaires among SANG soldiers (Jeddah, 2009)

**Questionnaire questions**	**Intervention**		**Questionnaire questions**	**Intervention**	
	**Pre**	**Post**		**Pre**	**Post**
**Knowledge of HBV nature**			**Knowledge of HBV transmission**		
HBV is an infectious disease	133 (33.3%)	391 (97.8%)	Using other person's toothbrush	133 (33.3%)	394 (98.5%)
Organs affected by HBV	213 (53.3%)	398 (99.5%)	Dentist tools	171 (42.8%)	398 (99.5%)
The type of causative microorganism	142 (35.5%)	394 (98.5%)	Razor	165 (41.3%)	397 (99.3%)
Infected person will remain infected for life	70 (17.5%)	252 (63.0%)	Surgery tools	190 (47.5%)	397 (99.3%)
Can transmit through the family members	134 (33.5%)	351 (87.8%)	Circumcision tools	150 (37.5%)	396 (99.0%)
HBV is easily transmitted than AIDS	53 (13.3%)	361 (90.3%)	Cupping " Hijamah" tools	171 (42.8%)	393 (98.3%)
Can be diagnosed from external appearance	96 (24.0%)	302 (75.5%)	Acupuncture needles	161 (40.3%)	392 (98.0%)
When to call the disease as chronic	55 (13.8%)	358 (89.5%)	Nails clipper	62 (15.5%)	395 (98.8%)
The percentage of chronic disease	7 (1.8%)	244 (61.0%)	Sewak (tooth cleaner)	113 (28.3%)	397 (99.3%)
HBV has complications	121 (30.3%)	378 (94.5%)	Ear or nose piercing	67 (16.8%)	387 (96.8%)
Most of the liver tumors are caused by HBV	70 (17.5%)	333 (83.3%)	Tattooing	81 (20.3%)	391 (97.8%)
Infected person may develop liver cirrhosis	128 (32.0%)	390 (97.5%)	**Knowledge of HBV preventive and control**		
Infected person may develop cancer	76 (19.0%)	376 (94.0%)	Availability of any protection against HBV	195 (48.8%)	390 (97.5%)
HBV could lead to death	142 (35.5%)	378 (94.5%)	Availability of vaccine against HBV	202 (50.5%)	386 (96.5%)
**Knowledge of HBV transmission**			Pregnant women should do HBV screening	162 (40.5%)	391 (97.8%)
Blood	232 (58.0%)	399 (99.8%)	Family members of patients should do investigations for HBV	292 (73.0%)	394 (98.5%)
Sexual intercourse	159 (39.8%)	398 (99.5%)	Availability of medical treatment	184 (46.0%)	364 (91.0%)
From infected mother to the fetus	120 (30.0%)	392 (98.0%)	Possibility of complete cure	17 (4.3%)	272 (68.0%)
From asymptomatic infected person	99 (24.8%)	336 (84.0%)	**Attitude towards HBV patients**		
Breast feeding	31 (7.8%)	358 (89.5%)	Dealing with infected household person	181 (45.3%)	279 (69.8%)
Shaking hands	166 (41.5%)	376 (94.0%)	Isolating infected person from work/daily activity	135 (33.8%)	373 (93.3%)
Hugging	124 (31.0%)	363 (90.8%)	Using toilet after an infected person	39 (9.8%)	253 (63.3%)
Contaminated clothes	54 (13.5%)	369 (92.3%)	**Practices of HBV patients**		
Sneezing	52 (13.0%)	376 (94.0%)	Eating food with his/her family	112 (28.0%)	371 (92.8%)
Coughing	51 (12.8%)	0 (0.0%)	Sharing the eating tools with his/her family	49 (12.3%)	306 (76.5%)
Eating food prepared by an infected person	60 (15.0%)	346 (86.5%)	Kissing his/her children	114 (28.5%)	384 (96.0%)
Sharing food with an infected person	95 (23.8%)	337 (84.3%)	Shaking hand of his/her children	186 (46.5%)	386 (96.5%)
Water drinking	43 (10.8%)	267 (66.8%)	Helping injured subject with open wound	134 (33.5%)	293 (73.3%)
Using syringes or needles	209 (52.3%)	396 (99.0%)			

P-values of Chi Square tests comparing pre- and post-intervention questionnaires were <0.001 for all questions.

Similar to the general knowledge questions, attitude and practice questions also significantly improved after intervention (p<0.001 for all). Those who expressed the correct attitude in cautiously dealing with an infected household person and not supporting isolation of that infected person from work or daily activity increased from 45.3% and 33.8%, respectively before intervention to 69.8% and 93.3%, respectively after intervention. Those who expressed their acknowledgement to some behaviors of HBV patients such as eating food with his/her family and shaking hand of his/her children increased from 28.0% and 46.5%, respectively before intervention to 92.8% and 96.5%, respectively after intervention. Likewise those who denounced that HBV patients can help injured subject with an open wound increased from 33.5% before intervention to 73.3% after intervention.

**Figure 1:**
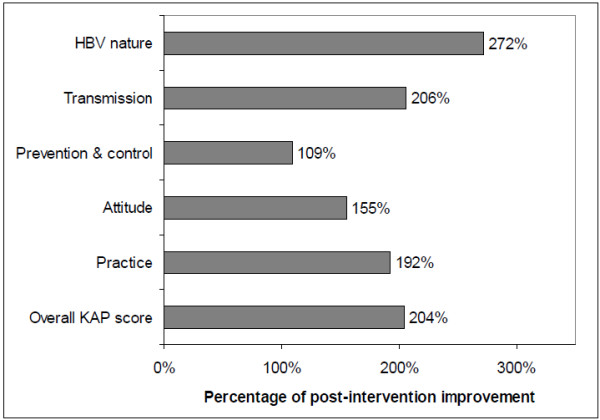
**Percentage of post-intervention improvement of mean overall knowledge, attitude, and practice (KAP) score and its components’ scores compared to pre-intervention scores among SANG soldiers (Jeddah, 2009).** P-values using Wilcoxon signed rank tests for all scores’ improvements were <0.001.

The median (and IQR) of overall KAP score significantly increased from 16 (6–26) before intervention to 53 (50–55) after intervention (p-value of Wilcoxon signed rank test <0.001) (Table 1). This represented more than a 200% increase of post-intervention mean overall KAP score (Figure 1). The improvement of KAP components’ scores were highest with HBV nature (272%) and lowest with prevention and control (109%) (Figure 1). The improvement of post-intervention overall KAP score was evident irrespective (after stratification) of age groups, education level, marital status, monthly income, job type or rank, duration of service, and history of HBV vaccine (Table 1). Using Mann–Whitney test or Kruskal Wallis test, both pre- and post-intervention, overall KAP scores were significantly associated (p-value <0.05) with higher educational levels, higher monthly income (>8000 Saudi Riyals), administrative jobs, and higher job ranks but not marital status nor duration of service. Additionally, a pre-intervention overall KAP score was significantly associated with history of HBV vaccine (p=0.039) (Table 1).

## Discussion

The current study examined KAP of HBV infection among military personnel before and after educational intervention among SANG soldiers. We are reporting low pre-intervention knowledge levels. Common methods of HBV transmission such as blood transfusion, sexual intercourse, and childbirth acknowledged by our population before educational intervention were limited (58%, 40%, and 30%, respectively) and were comparable to those reported by Saudi dental patients (50-52%, 32-38%, and 33-41%, respectively). However, the level of knowledge in our SANG population was considerably lower than reported in the non-Saudi population with a relatively higher risk of HBV [17-19]. For example sexual intercourse and childbirth as a potential source of HBV were recognized by 69% and 83% (respectively) of Vietnamese American compared to 40% and 30% (respectively) of SANG soldiers [18]. Similarly, blood transfusion and sexual intercourse were recognized by 93% and 68% (respectively) of Egyptian barbers and their clients compared to 58% and 40% (respectively) of SANG soldiers [17]. Some of these differences may be understood given the highly conservative nature of the Saudi community who find it embarrassing to openly discuss issues such as sexually transmitted diseases or safe sex with partners. Moreover, the lack of health education campaigns targeting important health problems in military personnel may also have contributed to this serious absence of knowledge. The low level of HBV awareness observed among our SANG population before the intervention may reflect a similarly low level of HBV awareness in the general public in SA [12] which may be contributing to the endemicity of HBV.

The study results showed marked improvement of overall KAP scores and their components’ scores after educational intervention. The observed post-intervention improvement among SANG soldiers was evident irrespective of socio-demographic characteristics and history of HBV vaccine intake. Similar results were reported using different educational methods in different populations [20,21]. A significant improvement in the knowledge of HBV prevention was observed in five Asian-American groups who completed self-administered tests before and after receiving lectures on hepatitis B prevention [20]. Additionally, in a randomized trial among Chinese Americans/Canadians lay health worker, those who received standard HBV audiovisual and printed educational materials were able to recognize common methods of HBV transmission after 6-month from randomization significantly better than controls [21]. Similarly, a marked improvement (from 24% to 84%) in knowledge of hepatitis C transmission was observed in secondary school students in France after an educational slide show. The improvement was independent of gender, age and geographical area [22]. Interestingly, the knowledge sub-score for HBV preventive and control in our SANG population had the poorest improvement score. This could simply be due to the higher level of pre-intervention knowledge of preventive and control compared to the disease nature, for example, which was the highest to improve.

The overall KAP score in our SANG population both before and after educational intervention was significantly associated with higher educational levels, higher monthly income, administrative jobs, and higher job ranks. These characteristics may be inter-correlated; for example those with higher education have higher-paid jobs and are more administratively inclined. HBV awareness in many populations was reported to correlate with the level of education [12,13,23,24] and higher income [12,24]. Those with higher education or higher income probably have more and better access to educational resources such as internet as well as preventive care including physician advice.

The current study had many strengths including targeting a military population that has been ignored in previous KAP studies, large sample size (N=400), lower level of non-participation (4%), and the absence of post-intervention drop-outs that was shown in other studies [21]. Nevertheless, we acknowledge the limitations. Our findings cannot be projected to the general population as the military population is likely to have different exposures and is inherently more disciplined, as indicated by the high participation rate and completeness of post-intervention survey. However, our military population had a level of pre-intervention knowledge similar to other members of the SA population [12]. Additionally, our design assessed the immediate post-intervention KAP level and, therefore, cannot be extended to long-term improvement of KAP level. Future studies within the military population may need to focus on long-term sustainability of such improvement.

### Conclusion

In conclusion, we are reporting a low level of HBV awareness among the Saudi military population. The study confirms the need and the effectiveness of focused multifaceted educational campaigns in improving KAP among the military population. Targeting military recruits may ensure earlier education and, most likely, protection.

## Competing interests

All authors have no competing interests.

## Authors' contributions

MT, designed the study, overviewed implementation, and drafted the manuscript. HB, helped in drafting and critical review of the manuscript. ZM, participated in the study design and helped critical review of the manuscript. YM, participated in the study design and helped critical review of the manuscript. AI, participated in the study design and supervised statistical analysis. AA, participated in the study design and helped critical review of the manuscript. AT, supervised the study design and implementation and helped to draft the manuscript. All authors read and approved the final manuscript.
